# Evaluating Ovarian Cancer Risk–Reducing Salpingectomy Acceptance: A Survey

**DOI:** 10.1158/2767-9764.CRC-24-0566

**Published:** 2025-01-30

**Authors:** Alexandra Lukey, Ramlogan Sowamber, David Huntsman, Celeste Leigh Pearce, A. Fuchsia Howard, Rafael Meza, Michael R. Law, Minh Tung Phung, Gillian E. Hanley

**Affiliations:** 1 Faculty of Medicine, School of Population and Public Health, University of British Columbia, Vancouver, Canada.; 2 Department of Obstetrics and Gynecology, Faculty of Medicine, University of British Columbia, Vancouver, Canada.; 3 BC Cancer Research Institute, Vancouver, Canada.; 4 Department of Medical Genetics, Faculty of Medicine, University of British Columbia, Vancouver, Canada.; 5 Department of Pathology and Laboratory Medicine, Faculty of Medicine, University of British Columbia, Vancouver, Canada.; 6 Faculty of Applied Sciences, School of Nursing, University of British Columbia, Vancouver, Canada.; 7 Department of Epidemiology, School of Public Health, University of Michigan, Ann Arbor, Michigan.; 8 Department of Population Health Sciences, University of Wisconsin–Madison, Madison, Wisconsin.

## Abstract

With evidence that salpingectomy is effective in preventing high-grade serous carcinoma, it is time to consider offering this procedure to people at higher-than-average lifetime risk for ovarian cancer, despite not having a pathogenic genetic variant that increases the risk for ovarian cancer. This targeted approach has potential to be effective at reducing ovarian cancer incidence, and unlike opportunistic salpingectomy, it is focused on people with an increased lifetime risk of ovarian cancer. However, the acceptability of this approach within the population of potential patients remains unknown. We conducted an online survey of adults in British Columbia, Canada, who were defined as “at risk” for ovarian cancer (i.e., people born with ovaries). Participants completed a questionnaire on demographics, ovarian cancer risk and protective factors, concerns about risk-reducing salpingectomy (RSS), and the risk they considered high enough to warrant RRS. We included 211 participants. Among these participants, 42% (*n* = 88) indicated that they would consider RRS at any lifetime risk or any risk above the population average. Another 20 participants chose risks between 1.5% and 4% for a cumulative 51% of the sample choosing risks below thresholds for oophorectomy. In contrast, 6% (*n* = 12) indicated that they would not consider the procedure at any risk level. None of the factors collected were associated with the likelihood that a person would find RRS acceptable. Overall, our participants showed broad interest in RRS as an ovarian cancer prevention strategy. These results suggest that there would likely be uptake if RRS was offered.

**Significance:**

This study found that many participants were willing to consider RRS to prevent ovarian cancer. Further research on RRS should be undertaken to understand how this can be best used for ovarian cancer prevention.

## Introduction

Epithelial ovarian cancer accounts for 4.7% of cancer deaths globally and continues to have 5-year survival rates of below 50% (1–4). This poor survival can be attributed to the origins and biology of this cancer, which explains the failure of ovarian cancer early detection approaches, combined with vague and nonspecific symptoms that generally arise when the disease is already in advanced stages (5). One of the most promising routes to reducing the incidence of ovarian cancer is the prevention strategy called opportunistic salpingectomy (OS). OS is the removal of the fallopian tubes during another planned pelvic surgery, most commonly during hysterectomy, or as an alternative to tubal ligation (6, 7). Salpingectomy was found to significantly reduce the risk of high-grade serous carcinoma (HGSC; ref. 8), the histotype accounting for 80% to 90% of ovarian cancer deaths (9–11). It is also possible that salpingectomy reduces the risk of clear-cell and endometrioid ovarian cancer histotypes, although this evidence is not conclusive (12). Historical studies show a relative risk reduction of bilateral salpingectomy on any type of epithelial ovarian cancer in the range of 35% to 64% (7, 13–15). Thus far, evidence has also been reassuring that salpingectomy alone does not affect the age of menopause (16, 17).

With over a decade of study of OS, global uptake is on the rise, with at least nine countries having formal recommendations for OS (18–20). In Canada between 2017 and 2020, there were 35,228 salpingectomies done alongside hysterectomy and another 25,633 salpingectomies done for the purpose of sterilization (20). The strong evidence of the effectiveness of OS in preventing HGSC and the growing acceptance of OS within obstetrics and gynecology has resulted in interest in expanding the “opportunities” to perform salpingectomy beyond gynecologic surgeries (6, 21, 22).

It is important to note that OS differs from the prevention offered to individuals with high-risk genetic variants, such as *BRCA1* and *BRCA2*. Risk-reducing bilateral salpingo-oophorectomy (RRBSO) remains the recommended prevention strategy for these individuals as it reduces all-cause mortality by 70% in this patient population (23). RRBSO differs significantly from OS because of the hormonal consequences of oophorectomy (i.e., removal of the ovaries), particularly in premenopausal people (23). Offering salpingectomy followed by oophorectomy to individuals with *BRCA* or similar mutations as a means of reducing cancer risk and preventing the medical consequences of surgical menopause is being explored in clinical trials (24). So although any person undergoing a pelvic surgery may be offered OS and people at very high lifetime risk are currently offered RRBSO, it is not a standard practice to intervene to reduce risk for ovarian cancer in people who are at a considerably increased risk of developing ovarian cancer but not at a sufficiently high lifetime risk to warrant the receipt of RRBSO.

Many factors increase an individual’s lifetime risk for ovarian cancer, including common susceptibility variants, which can explain about 6% of the heritability of ovarian cancer (25), as well as differences in known risk and protective factors. Although rare variants in well-known high and moderate penetrance susceptibility genes (*BRCA*, *BRIP1*, *PALB2*, *RAD51C*, and *RAD51D*) explain about 40% of the inherited component of ovarian cancer (26, 27), polygenic risk scores (PRS; a weighted sum of the number of risk alleles carried for a specific set of variants) may provide an opportunity to refine risk stratification for the general population and in carriers of rare moderate- or high-risk alleles. Although PRS alone are not yet recommended for clinical decision-making, when combined with other well-known risk and protective factors for ovarian cancer, tools exist to estimate a person’s lifetime risk of developing ovarian cancer, such as CanRisk (26, 28). People at sufficiently increased risk, such as those with significant family histories, but who do not have the highly penetrant pathogenic variants described above that would make them eligible for RRBSO, could be offered risk-reducing salpingectomy (RRS), defined as salpingectomy for the primary indication of cancer prevention performed as a stand-alone surgery rather than opportunistically. The appropriate lifetime risk for ovarian cancer to warrant RRS remains to be determined. However, given that salpingectomy has comparatively few long-term negative health consequences compared with salpingo-oophorectomy (29, 30), the RRS risk threshold is likely to be lower than that for RRBSO, which is currently recommended to be about 4% to 5% lifetime risk (31–34). It is also important to state that what we are proposing differs from salpingectomy with delayed oophorectomy, which is currently under study (https://clinicaltrials.gov/, NCT02760849 and NCT02321228, accessed May 15, 2024). The target population for RRS with delayed oophorectomy would be considered the group that, at this time, meets the guideline criteria for oophorectomy. In contrast, this work aims to understand the acceptability of RRS for those who have elevated risk but are below the threshold for oophorectomy.

RRS represents a notable difference from what is now recommended and offered to patients, and thus, patient perspectives related to RRS in the absence of a pathogenic variant warrant exploration. Whereas satisfaction with an intervention can only be measured retrospectively, acceptability can be measured prospectively (35). Therefore, we assessed the acceptability of RRS from the perspective of people from the general public and how the perceived acceptability compares with the proposed threshold of lifetime risk to recommend RRBSO. In addition, our secondary objectives were to explore whether individual factors influence acceptability and, finally, to understand patients’ concerns about RRS.

## Materials and Methods

We completed a province-wide, nonprobability cross-sectional online survey between August and November 2023. We recruited English-speaking adults in British Columbia (BC), Canada, who were defined as at risk for ovarian cancer (i.e., people born with ovaries) and who had no current intentions for future pregnancies. Methods are reported following the CHERRIES guidelines for online surveys (36). Exclusion criteria included (i) a prior gynecologic cancer; (ii) having previously undergone unilateral or bilateral salpingectomy or salpingo-oophorectomy; and/or (iii) a known pathogenic variant that increases risk (e.g., *BRCA1* or *BRCA2* variant). We set these exclusion criteria because we were interested in the opinions of participants who have not already undergone salpingectomy or a previous gynecologic cancer (experience naïve). We also did not want to include those who would be counseled to undergo RRBSO instead of RRS. We also excluded people that either intend or are unsure about their desire to become pregnant in the future because this would make them ineligible for salpingectomy, making prospective acceptability too distant to measure.

Participants were recruited throughout BC using several recruitment strategies to gather perspectives from people across the province. Recruitment strategies included physical posters, a social media campaign, and advertising on the REACH BC platform. REACH BC is a provincial nonprofit initiative that connects participants to research opportunities for which they are eligible. Physical posters were displayed in public areas across several regions in BC, including the Interior, Lower Mainland, and Vancouver Island. Finally, the social media campaign included paid advertising and unpaid social sharing on Instagram and Facebook of study recruitment materials.

Participants were prescreened for eligibility criteria using a presurvey before being invited to participate. We offered a $25 gift card as an incentive for participation. To ensure that participants who responded to the survey were not duplicates or bots, we used captcha and required participants to provide us with a physical address to send their gift cards.

After screening and informed consent were complete, we provided participants with a one-page information pamphlet, created by the study team and written for a lay audience about ovarian cancer and salpingectomy to ensure that people understood the research topic (Supplementary Material S1). Participants then completed an online questionnaire on demographics, risk and protective factors for ovarian cancer, and a short survey that asked questions about their thoughts on RRS for ovarian cancer prevention, including what lifetime risk they considered actionable. The survey was developed through consultation among the study team, and the participants were able to skip any question. Consent and data were collected using REDCap, which is a secure web application specifically created for managing research data (37, 38). We obtained ethics approval for this study through a harmonized review by The University of British Columbia (certificate number: H22-03281).

We used SAS and Excel for descriptive analysis (perceived acceptability, warranted lifetime risk, and concerns; ref. 39). We plotted histograms of the lifetime risk score that participants chose as warranting RRS and for the most common concerns related to RRS. Following this, we created a binary variable based on the lifetime risk scores, categorizing the participants as indicating lifetime risks of less than or greater than 4% to warrant RRS (the lifetime risk they would likely be offered RRBSO rather than RRS; ref. 34).

Survey responses were analyzed using logistic regression to assess the association of personal characteristics with interest in receiving RRS. Due to power issues, we collapsed some variables, such as ethnicity, household income, and gender identity, into a small number of categories. We applied hot deck imputation to address missing covariate data, in which missing values for a respondent (known as a recipient) were substituted with observed values from another respondent (known as a donor) who had complete data (40). We also repeated all analyses with a complete case sensitivity analysis. The free-text response option for the question about the greatest concerns was analyzed thematically, although qualitative analysis was not the focus of this study.

### Data availability

The data generated in this study are not publicly available because of participant privacy concerns but are available upon reasonable request to the corresponding author for verification purposes.

## Results

A total of 246 people consented to participate in the survey. Of this initial sample, 29 people who consented did not complete the survey and 6 people who completed the survey had missing data for the outcome (lifetime risk that would warrant RRS) so were excluded. One person started but did not submit their survey. Therefore, our final analytic sample consisted of 211 participants, of whom 67 (32%) had some missing covariate data that we imputed. Variables with missing data that were imputed were education, gender, income, live births, partnership status, rurality, and race/ethnicity. Variables included in the imputation model were all the variables listed previously, along with variables without missing data (age and menopause status) and the outcome variable. The variable with the highest level of missingness was the number of live births, which had a missingness of 14%. Our sample had an average age of 44.1 years (SD, 13.27), 92% were cisgendered women, 78% were White, and 70% had an undergraduate degree or higher (Table 1).

**Table 1:** Sample characteristics of 211 participants

Characteristic	Overall (*N* = 211)
Age, years [mean (SD)]	44.10 (13.27)
Education, *n* (%)	
Less than high school up to college	61 (28.9)
Undergraduate	70 (33.2)
Postgraduate	78 (37.0)
Missing	2 (0.9)
Gender, *n* (%)	
Cisgendered female	195 (92.4)
Trans men and/or nonbinary	10 (4.7)
Missing	6 (2.8)
Income, *n* (%)	
<$60,000	46 (21.9)
$60,000–$100,000	50 (23.6)
>$100,000	95 (45.0)
Missing	20 (9.5)
Live births [mean (SD)]	0.97 (1.14)
Partnership status, *n* (%)	
Partnered	136 (64.5)
Unpartnered	71 (33.6)
Missing	4 (1.9)
Rurality, *n* (%)	
Small center	37 (17.5)
Medium center	26 (12.3)
Large center	144 (68.2)
Missing	4 (1.9)
Race/ethnicity, *n* (%)	
Person of color	37 (17.5)
White	165 (78.2)
Missing	9 (4.3)
Menopause, *n* (%)	211
Premenopause	139 (65.9)
Postmenopause	72 (34.1)

**Figure 1:**
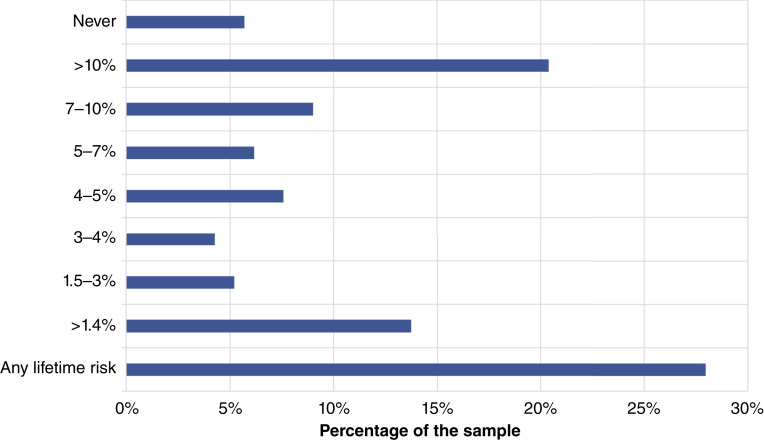
Bar graph of the lifetime risk of ovarian cancer that participants thought warrants RRS. Data from the survey, presented as the percentage of participants who chose each option.

Of the 211 participants included in the analysis, 28% (*n* = 59) indicated that they would consider RRS at any lifetime risk and another 14% (*n* = 29) indicated that they would consider RRS at any risk above the population average of 1.4%. An additional 20 participants chose lifetime risks below 4% to 5%, for a cumulative 51% of the sample choosing risks lower than thresholds for RRBSO. Twelve participants (6%) indicated that they would not consider RRS at any risk level (Fig. 1).

When examining factors associated with the lifetime risk participants found RRS to be acceptable in the univariate model, no individual factors were significantly associated with reporting a higher lifetime risk to warrant RRS (>4% vs. <4%; Table 2). In the multivariate model, there were also no individual factors significantly associated with the lifetime risk of RRS participants thought was actionable. However, having less education than an undergraduate degree directionally increased the likelihood that participants chose a lifetime risk to warrant RRS less than 4% [adjusted odds ratio (aOR), 0.57; 95% CI, 0.30–1.08]. When examining the models in which only complete cases were used, there was no change in the significance or directionality of results (Supplementary Table S1).

**Table 2:** Logistic regression analysis of factors associated with accepting risk-reducing surgery (RRS) for ovarian cancer when the lifetime risk is less than 4%, compared with greater than or equal to 4%, using hot deck imputation

Characteristic	Univariate			Multivariate		
	OR	95% CI	*P* value	OR	95% CI	*P* value
Overall (*n* = 211)						
Age	1.00	0.98–1.02	0.88	1.00	0.98–1.02	0.90
Education						
Less than high school up to college	Ref.			Ref.		
Undergraduate to postgraduate	0.58	0.32–1.07	0.08	0.57	0.30–1.08	0.08
Income						
<$60,000	Ref.			Ref.		
$60,000–$100,000	0.96	0.44–2.08	0.91	1.03	0.45–2.28	0.94
>$100,000	0.96	0.49–1.87	0.90	1.12	0.52–2.41	0.78
Race/ethnicity						
Person of color	Ref.			Ref.		
White	1.27	0.64–2.52	0.49	1.16	0.58–2.32	0.69
Gender						
Transgender or nonbinary	Ref.			Ref.		
Cisgendered	0.95	0.27–3.39	0.94	1.09	0.28–4.20	0.90
Partner status						
Unpartnered	Ref.			Ref.		
Partnered	0.95	0.54–1.67	0.85	1.05	0.55–2.02	0.89
Number of live births	0.99	0.78–1.27	0.96	0.93	0.71–1.21	0.57

Multivariate model included age, education, income, race/ethnicity, gender, partner status, and number of live births.

Abbreviation: CI, confidence interval.

**Figure 2:**
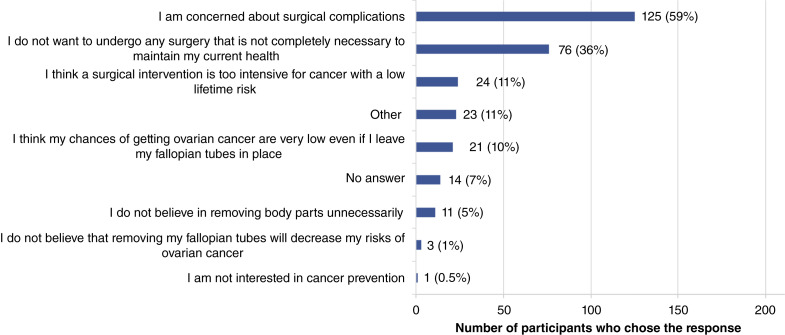
Bar graph of the most common concerns related to RRS from the survey. Cumulative percentage will add up to more than 100% as participants could choose multiple options.

Frequencies of concerns related to RRS are shown in Fig. 2. Of the 197 (14 people left the concerns question blank) people who cited a concern, the two most common concerns were the risk of surgical complications at 59% (*n* = 125) and the desire not to undergo surgery unless absolutely necessary at 36% (*n* = 76). Twenty-three (11%) participants entered other concerns in the free-text box. The most common of the “other” responses (7/23) indicated concerns related to undergoing general anesthesia but expressed that they would consider the procedure if it could be completed without the use of general anesthesia. The remaining “other” responses included concerns about recovery time after surgery, the desire for more information, and evidence about the long-term consequences of salpingectomy and the concern that they would change their mind about the desire for pregnancies in the future.

## Discussion

An increasing body of evidence suggests the effectiveness of salpingectomy for ovarian cancer prevention. Our results demonstrate that a sizable proportion of respondents in BC were open to considering RRS with a higher-than-average lifetime risk of ovarian cancer. Specifically, 51% of this sample would consider RRS at lifetime risks below 4% to 5%, the currently recommended threshold for RRBSO (32, 34). In this sample, we found no evidence of significant differences in the acceptability of RRS depending on different sociodemographic factors, suggesting that acceptance could be widespread. However, participants also cited concerns related to receiving RRS, most commonly related to the risks of surgical complications and a lack of desire to undergo preventive (rather than curative) surgery. This division between the constructs of the theoretical framework of acceptability, such as effective attitude (acceptable lifetime risk), compared with burden and ethicality constructs (concerns related to RRS), shows the importance of measuring different constructs of acceptability (35). When faced with whether or not to undergo RRS, patients may, in theory, approve of the intervention but will weigh the benefit against the real or perceived burdens of the intervention, or in the context of surgery, the perceived safety and risk (41).

Although this study is, to the best of our knowledge, the first to describe the patient’s acceptability of RRS, our work aligns with the previous work related to patient acceptance and satisfaction with respect to other risk-reducing surgeries. A recent systematic review and meta-analysis, which included risk-reducing surgeries (RRBSO, risk-reducing early salpingectomy and delayed oophorectomy, and risk-reducing mastectomy) for people at a high risk for breast and ovarian cancers, evaluated 34 studies (42). This study found detrimental changes in the quality of life after a risk-reducing surgery that were related to menopause (in which people received oophorectomy) and body image (risk-reducing mastectomy). However, as salpingectomy alone does not result in surgical menopause (16, 43) and there are no changes in physical appearance after salpingectomy, we would expect a lesser impact on the quality of life for people undergoing RRS. Thirteen studies found a decrease in sexual pleasure after RRBSO but no change in sexual function after salpingectomy alone (42). Another recent study followed 22 people with *BRCA1* and *BRCA2* variants who had received salpingectomy alone for 3 years to assess their feelings and perceptions of risk and reward. All participants in this small study were satisfied with their decision to undergo salpingectomy (44). Although this group is at a much higher risk for ovarian cancer, their satisfaction with salpingectomy may be relevant to the population who would receive RRS, particularly as relates to the lack of important changes to their quality of life based on undergoing salpingectomy alone. The existing evidence and our data thus suggest that although we could expect similar outcomes related to acceptability and satisfaction, we would not expect to see the adverse effects related to body image and sexual health that are related to RRBSO and risk-reducing mastectomy.

Relatedly, there is some research on the patient acceptability of OS. It has been shown that acceptance rates can be as high as 96% when patients are counseled on OS (45). Gelderblom and colleagues explored the perspectives of patients in a mixed-method study. They found barriers to patients deciding about OS similar to those we found in our study. In relation to OS, the most common obstacles to OS were a lack of knowledge about the surgery, not wanting to remove healthy organs, and concern about surgical complications (43). However, people undergoing OS are already having surgery for another indication. Given that RRS would be a stand-alone surgery, which always confers some degree of risk, it is reassuring that individuals in our survey were carefully weighing the minimum lifetime risk for RRS to be warranted.

Although OS has been called the new “*de facto*” standard in some jurisdictions (19), targeting individuals who are at a higher-than-average lifetime risk for ovarian cancer, despite not having a pathogenic variant, will likely result in a much lower number needing treatment to prevent an HGSC. Incorporating RRS alongside the opportunistic model of prevention is likely to produce a much greater impact on the incidence of HGSC in the future. Although this work is important in outlining the prospective acceptability of RRS, more work is needed before implementing this clinically.

Most pressing, perhaps, is the need for a clear understanding of the complication rate of RRS. This is necessary to weigh the individual and economic risks to benefit the profile of the procedure. It is also important for patient counseling in the case in which a patient is considering the procedure but also weighing the acceptability of safety and risk of the intervention (35, 41). Although work has been done on the safety of OS compared with hysterectomy alone or tubal ligation (29, 46–48), RRS would constitute a different population and primary motivation for surgery and therefore should be evaluated independently.

Also, if RRS became a standard practice, much research would be needed with regard to optimal anesthesia. As highlighted by some of our participants, general anesthesia would pose a barrier for some patients. One systematic review compared local anesthesia with conscious sedation versus general anesthesia for female laparoscopic sterilization and found similar outcomes with regard to complications and operating time but superior patient satisfaction and recovery time for those who received conscious sedation compared with general anesthesia (49).

Furthermore, although our article explored RRS interest from the viewpoint of the general public, we also need to explore providers’ perspectives (50, 51). Taking into account both perspectives is important when deliberating how to proceed with salpingectomy as a prevention strategy. Similarly, more work will need to be done to define acceptable thresholds for offering RRS. Although some in our study stated that they would consider RRS at “any” lifetime risk, the recommendation to remove the fallopian tubes in all people desiring no future pregnancies is unadvisable because of the risks to the individual’s health of undergoing even a minor and safe procedure like salpingectomy (33, 52). Furthermore, although validated tools to estimate the lifetime risk of ovarian cancer such as CanRisk exist (28), little is known about what population-based testing for ovarian cancer would entail. For instance, more work is needed to understand attitudes, costs, and potential unintended consequences of such a population-based testing program.

Additionally, from the perspective of the health system, it is unlikely that RRS for people at an average lifetime risk would be a priority from a health system planning perspective, compared with other priority procedures such as joint replacements (53). Therefore, similar to RRBSO (54), clear thresholds of when RRS ought to be recommended to this “moderate risk” population are needed if patients were to be counseled adequately. This will continue to be clarified as our understanding of the role of PRS and their applications to clinical practice improves. At the time of writing, PRS alone have not been sufficiently validated to be used in clinical management outside of trials (55).

### Strengths and limitations

An important limitation of this work is that these results are based on a nonprobability sample and may not be generalizable. Although our sample included a range of ages, genders, races/ethnicities, rurality, education, and incomes, our sample is more highly educated than the provincial averages. Although 57.5% of people in Canada have college or university degrees (56), only 10.4% of our sample had high school or less education. This may have implications with regard to differences in risk literacy between our sample and the general population. Our sample also had fewer live births than would be expected based on the BC population average of 1.1 child per woman in 2022 (57). Furthermore, our sample had a higher proportion of nonbinary and transgender people, 4.7% compared with 0.44%, according to data from the BC 2021 Census (58). The sample had fewer people from racialized groups who comprised 17.5% of our sample, compared with the provincial composition, which is 34% (59). Rurality was slightly higher than the provincial average, with 17.5% of our sample living in small centers compared with the 12.7% provincial average of people living rurally (60). Finally, our sample, on average, had a higher household income, with 45% of the sample stating household incomes above $100,000 compared with the provincial median household income of $76,000 (61).

Our sample may also differ from the underlying population in unobserved ways, putting the results at risk for selection bias, such as having a higher interest in health and preventative behaviors. Furthermore, due to the exploratory nature of the study, we collapsed certain sociodemographic categories, such as gender and race. However, this may obscure differences in these identities. Our results must also be interpreted within the context of Canada’s publicly funded healthcare system in which all surgical and physician services are covered for all Canadians (62). In settings where surgery is privately insured or paid for out-of-pocket, cost would be an important, potentially prohibitive factor. Finally, although acceptability can be measured prospectively (35), in this study, our participants were not offered salpingectomy, so we cannot confirm whether this acceptance would translate to action. One strength of the study is that although not randomly sampled, we used various recruitment methods, allowing us to reach a wide range of people, such as those living in rural areas, who are often not included in research. However, due to the varied recruitment methods, we can also not reliably calculate a survey response rate.

### Conclusions

The acceptance of RRS as a prevention strategy for ovarian cancer in a sample of individuals at risk for ovarian cancer from the general population was high, with interest in undergoing RRS appropriately varying by warranted lifetime risk. Thus, much research is warranted to explore the safety and feasibility of offering RRS as a stand-alone surgery to people who are at a higher lifetime risk than the average population but who should not undergo RRBSO. A particular focus should be on determining the complication rate of the procedure, which our study highlights as a priority both from the patient’s and health system’s perspectives.

## Supplementary Material

Supplementary Material 1 Education pamphlet for participants

Supplementary Table 1 Supplementary Table 1. Logistic regression analysis of factors associated with accepting risk-reducing surgery (RRS) for ovarian cancer when lifetime risk is less than 4% compared to greater or equal to 4%, using complete case analysis
