# Supplementary material for: Evaluating Ovarian Cancer Risk–Reducing Salpingectomy Acceptance: A Survey
Source: Cancer Res Commun. 2025 Jan 30;5(1):187–94. doi: 10.1158/2767-9764.CRC-24-0566 (PMC11780486; doi:10.1158/2767-9764.CRC-24-0566)
Supplement: Supplementary Material 1 — Education pamphlet for participants [file crc-24-0566_supplementary_material_1_suppsm1.pdf]

WHAT YOU NEED TO KNOW

ARISE STUDY

What is ovarian cancer?

Ovaries are part of the female reproductive system and are responsible for releasing eggs and hormones such as estrogen and progesterone. Ovarian cancer happens when cells start multiplying abnormally, causing damage, and potentially spreading to other places in the body. Unfortunately, there is currently no effective way to screen for ovarian cancer, and it is a deadly disease with only a 50% survival rate beyond five years. On average, people without a genetic variant that increases their risk of ovarian cancer are at an 1.4% lifetime risk of getting ovarian cancer. That means on average about 1 to 2 people out of 100 may develop ovarian cancer if they do not take any preventive measures.

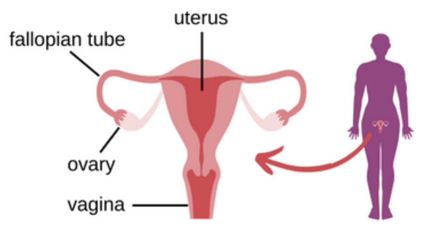

Why would fallopian tubes be taken out?

The fallopian tube is a tube that carries the egg from the ovaries to the uterus. Its main function is to help with fertility, and it does not have any other known roles in the body. Recent studies have shown that most high-grade serous ovarian cancers, which make up 70% of all ovarian cancers, actually start in the fallopian tube, then spread to the ovary. Removing the fallopian tubes through surgery can significantly reduce the risk of developing this type of cancer because it removes the tissue where these cancers typically start.

Why are we doing this research?

We now have evidence that salpingectomy is safe and early research suggests salpingectomy can lower the risk of developing ovarian cancer. Currently, we only recommend salpingectomy when people are having other pelvic surgery. Continued...

Page 2

WHAT YOU NEED TO KNOW

ARISE STUDY

Why are we doing this research?

Now, we want to explore how people feel about undergoing salpingectomy as a stand-alone surgery targeted towards individuals with a lifetime risk for ovarian cancer that is well above average. This work will provide vital information about how we can best prevent as many ovarian cancers as possible.

Why would we not remove fallopian tubes and ovaries?

The ovaries create hormones that are necessary for the body, especially for women before menopause. However, it's uncertain if removing the ovaries after menopause can lead to long-term health problems. Removing the ovaries before menopause can cause an increased risk of heart disease and osteoporosis (weakening of the bones). However, research suggests removing the fallopian tubes doesn't significantly affect the hormone production. Therefore, taking out the fallopian tubes can reduce the risk of ovarian cancer without raising the risks of heart disease and osteoporosis.

What are the risks and recovery time for salpingectomy?

Most salpingectomies are done using minimally invasive surgical techniques (laparoscopy, which is a surgical procedure that allows a surgeon to perform surgery without making large cuts in the skin), making it a low-risk surgery with a fast recovery time. This surgery is usually done under general anesthesia, and like any surgery, there are some risks. The most common risks of the surgery include bleeding, infection, pain, and reactions to the drugs you're given during surgery. Salpingectomy is also a permanent birth control, similar to tubal ligation (getting your tubes tied). On average, the total operation time is 70 minutes, and the length of stay in the hospital for a laparoscopic salpingectomy is 1/3 of a day. Previous research has shown about 1% of patients will be readmitted to the hospital following salpingectomy, and significantly fewer than 1% will experience an important surgical complication (requiring a blood transfusion). Most people leave the hospital the same day they have the surgery and can go back to their normal activities after several days. People who live in regions without access to gynecologists may need to travel to receive salpingectomy. Salpingectomy for sterilization is considered an elective surgery, and thus relevant wait times will apply (the length of which will depend on regional access to OR time).
