# Supplementary material for: Evaluating Ovarian Cancer Risk–Reducing Salpingectomy Acceptance: A Survey
Source: Cancer Res Commun. 2025 Jan 30;5(1):187–94. doi: 10.1158/2767-9764.CRC-24-0566 (PMC11780486; doi:10.1158/2767-9764.CRC-24-0566)
Supplement: Supplementary Table 1 — Logistic regression analysis of factors associated with accepting risk-reducing surgery (RRS) for ovarian cancer when lifetime risk is less than 4% compared to greater or equal to 4%, using complete case analysis [file crc-24-0566_supplementary_table_1_suppst1.docx]

*Supplementary Table 1. Logistic regression analysis of factors associated with accepting risk-reducing surgery (RRS) for ovarian cancer when lifetime risk is less than 4% compared to greater or equal to 4%, using complete case analysis*

|  | Univariate | | | Multivariate | | |
| --- | --- | --- | --- | --- | --- | --- |
| Characteristics | Odds ratio | 95% CI | P Value | Odds ratio | 95% CI | P Value |
| Overall (n = 144) |  |  |  |  |  |  |
| Age | 1.00 | 0.98-1.03 |  | 1.01 | 0.98-1.04 | 0.68 |
| Education |  |  |  |  |  |  |
| Less than high school up to college | REF |  |  | REF |  |  |
| Undergraduate to post-graduate | 0.73 | 0.35-1.53 | 0.40 | 0.64 | 0.29-1.42 | 0.27 |
| Income |  |  |  |  |  |  |
| <$60,000 | REF |  |  | REF |  |  |
| $60,000-$100,000 | 1.32 | 0.52-3.37 | 0.56 | 1.40 | 0.51-3.90 | 0.52 |
| >$100,000 | 1.34 | 0.61-2.96 | 0.46 | 1.58 | 0.58-4.31 | 0.37 |
| Race/ethnicity |  |  |  |  |  |  |
| Person of Colour | Ref |  |  | Ref |  |  |
| White | 1.41 | 0.61-3.26 | 0.43 | 1.66 | 0.66-4.14 | 0.28 |
| Gender |  |  |  |  |  |  |
| Transgender or Nonbinary | Ref |  |  | Ref |  |  |
| Cis-gendered | 1.28 | 0.28-5.92 | 0.75 | 0.69 | 0.13-3.64 | 0.66 |
| Partnered status |  |  |  |  |  |  |
| Unpartnered | Ref |  |  | Ref |  |  |
| Partnered | 1.10 | 0.56-2.13 | 0.79 | 0.89 | 0.40-2.00 | 0.79 |
| Number of live births | 0.89 | 0.67-1.19 | 0.43 | 0.83 | 0.60-1.16 | 0.28 |
| CI, confidence interval.  Multivariate model included age, education, income, race/ethnicity, gender, partner status and number of live births | | | | | | |
